# Physical, chemical, and biological routes of synthetic titanium dioxide nanoparticles and their crucial role in temperature stress tolerance in plants

**DOI:** 10.1016/j.heliyon.2024.e26537

**Published:** 2024-02-16

**Authors:** Nadiyah M. Alabdallah, Saleh M. Alluqmani, Hana Mohammed Almarri, Asla A. AL-Zahrani

**Affiliations:** a Department of Biology, College of Science, Imam Abdulrahman Bin Faisal University, P.O. Box 1982, 31441, City Dammam, Saudi Arabia; b Department of Physics. Faculty of Applied Science, Umm Al-Qura University, Makkah, 21955, Saudi Arabia; c Department of Physics, College of Science, Imam Abdulrahman Bin Faisal University, P.O. Box 1982, Dammam, 31441, Saudi Arabia; d Department of Chemistry, College of Science, Imam Abdulrahman Bin Faisal University, P.O. Box 1982, Dammam, 31441, Saudi Arabia; e Basic & Applied Scientific Research Center, Imam Abdulrahman Bin Faisal University, P.O. Box 1982, 31441, Dammam, Saudi Arabia

**Keywords:** Temperature stress, Environment, Titanium dioxide, Synthesis, Nanoparticles, Reactive oxygen species

## Abstract

Nanotechnology is attracting significant attention worldwide due to its applicability across various sectors. Titanium dioxide nanoparticles (TiO2NPs) are among the key nanoparticles (NPs) that have gained extensive practical use and can be synthesized through a wide range of physical, chemical, and green approaches. However, TiO_2_NPs have attracted a significant deal of interest due to the increasing demand for enhancing the endurance to abiotic stresses such as temperature stress. In this article, we discuss the effects of temperature stresses such as low (4 °C) and high temperatures (35 °C) on TiO_2_NPs. Due to climate change, low and high temperature stress impair plant growth and development. However, there are still many aspects of how plants respond to low and high temperature stress and how they influence plant growth under TiO_2_NPs treatments which are poorly understood. TiO_2_NPs can be utilized efficiently for plant growth and development, particularly under temperature stress, however the response varies according to type, size, shape, dose, exposure time, metal species, and other variables. It has been demonstrated that TiO_2_NPs are effective at enhancing the photosynthetic and antioxidant systems of plants under temperature stress. This analysis also identifies key knowledge gaps and possible future perspectives for the reliable application of TiO_2_NPs to plants under abiotic stress.

## 1 Introduction

Currently, the earth is experiencing a variety of environmental challenges, including the impacts of abiotic stresses. On average, abiotic stress can cause yield reductions of over 60% compared to record yields [1]. Rising global population, shrinking arable land, and increasing threats from climate change all put pressure on the need for novel approaches and strategies to boost yield potential under abiotic stress conditions like temperature. As global warming has continued, high and low-temperature stress have become key ecological limitations, threatening global food security. Both are considered abiotic stresses because they reduce crop yield at the morphological, physiological, biochemical, and cellular levels [2]. A rise of 1 °C in annual temperature led to decreases in crop yields ranging from 2.5% to 16% [3]. To meet the rising demand for food due to population growth, it's crucial to enhance plant tolerance to temperature stress and employ environmentally-friendly technologies.

Nanotechnology is an innovative method for enhancing the agriculture sector since it introduces new techniques for imparting tolerance to temperature stresses and increasing crop yield [4,5]. Nanoparticles (NPs) are molecules ranging in size from 1 to 100 nm. They possess a variety of physicochemical properties and exhibit increased reactivity and biological activity is dependent on their high surface energy and surface-to-volume ratio [6,7]. It has the ability to promote plant morphogenesis when used as herbicides, nanopesticides, and nanofertilizers, among other things, because they can efficiently release their material in appropriate proportions to target cellular organelles in plants [8]. Nonetheless, some NP potentials remain unknown due to a lack of mechanisms that have not been clarified or explored.

Titanium dioxide nanoparticles (TiO_2_NPs) play a significant role in various aspects of daily life. Consequently, it becomes imperative to attain a profound understanding of their potential toxicity and the detrimental consequences they may exert on both human health and the environment [9]**.** Several pieces of experimental evidence strongly indicate that exposure to TiO_2_NPs could be detrimental, leading to harmful health effects. These nanoparticles have been shown to cause damage to organs such as the spleen, liver, kidney, lungs, brain, and heart, as demonstrated in previous studies [10,11]. TiO_2_NPs have the greatest biological effect on plants, being helpful at low concentrations and toxic at high dosage. The impact of TiO_2_NPs on plants will vary depending on a number of variables, including the specifics of the environment, the variety of plants grown, and the concentrations of TiO_2_NPs used [12]. Similar to other nanomaterials, the concentration and characteristics of TiO_2_NPs are crucial to its application [13]. It has been documented that TiO_2_NPs enhances photosynthetic rate in crops as well as stimulating certain enzymes [14]. Despite being comparatively a new concept in agriculture compared to other sectors, nanomaterials have been found to increase crop yield as well as resistance to stress. Modern research indicates that certain metal oxides or nanoparticles not only enhance crop productivity but also do not create any metabolic imbalances [5].

By 2020, the world needed to increase wheat production by 60% to meet the growing demand for this vital crop [15]. High temperatures, however, affect wheat crop growth and productivity worldwide [2]. The effects of heat stress on plants are very complex and include changes in many biochemical and developmental responses [3]. Reactive oxygen species (ROS) are also produced during heat stress, which damage membranes and other system of cells as well as trigger stress responses [16,17]. For protection against oxidative damage, plants have developed complex antioxidant defense systems where the ascorbate-glutathione pathway plays a crucial role [18]. Nanoparticles induce oxidative stress in plants corresponding to generation of ROS. These ROS severely damage lipid membranes and other important macromolecules, including nucleic acids and proteins, which finally cause the death of cells in plants [19]. The ROS radicals act as signaling molecules, which stimulate plants' defense mechanisms against oxidative stress. Plants have developed antioxidant systems that help scavenge naturally generated reactive oxygen species (ROS) [20]. When nanoparticles are applied at appropriate concentrations, the activity of antioxidant enzymes is altered, enabling it to improve its resistance to heat stress. In addition, plants use both enzymatic antioxidants, such as SOD, CAT, APX, GPX, dehydroascorbate reductase (DHAR), and GR, as well as non-enzymatic antioxidants, including ascorbate, glutathione, thiols, and phenols [21]. Latef et al. [22] discussed three key mechanisms explaining the impact of titanium dioxide nanoparticles (TiO_2_NPs) on plants, both positive and negative. Firstly, TiO_2_NPs influence the balance of reactive oxygen species (ROS) signaling in plants, with both pro-oxidant and antioxidant properties. These nanoparticles are preferentially absorbed by chloroplasts, known sites of ROS production, affecting their function [23,24]. The overall effect on ROS levels and plant growth depends on TiO_2_NPs concentration, making TiO_2_NPs a regulator of plant growth through ROS-related signaling pathways [25,26]. The second mechanism involves TiO_2_NPs affecting plant nitrogen metabolism. When exposed to UV or sunlight, these nanoparticles can convert atmospheric nitrogen into nitrate. They also enhance the activity of nitrate reductase, an important enzyme in nitrogen assimilation. The third mechanism points out that the shape, size, and surface characteristics of TiO_2_NPs determine their availability to plants [27]. For instance, low concentrations of TiO_2_NPs (up to 5 ppm) can protect chickpea genotypes from cold damage [12]. However, higher concentrations may harm plants. Therefore, careful dosage and application methods are necessary before considering widespread use on crops.

However, there has not been a great deal of systematic scientific investigation into the effects of TiO_2_NPs on plants under low and high temperature stress conditions. As a result, we have compiled data from previous studies conducted between 2013 and 2023, with a particular emphasis on understanding how TiO_2_NPs influence a plants ability to withstand temperature stress. Additionally, we have detailed the characteristics and synthesis of TiO_2_NPs, while also demonstrating their role in triggering responses to low and high-temperature stress in plants.

## 2 Methods for the synthesis of TiO_2_ NPs

**Fig. 1:**
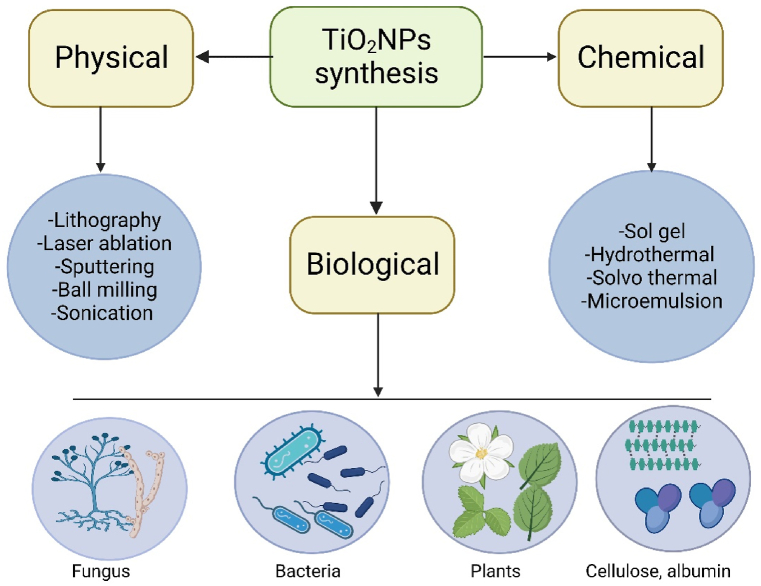
Titanium dioxide nanoparticles (TiO_2_NPs) synthesis through different physical, chemical and biological approaches. Created in Biorender.

TiO_2_NPs exhibits polymorphism, manifesting in three primary crystalline structures: anatase with a refractive index of 2.49, brookite with a refractive index of 2.58, and rutile with a refractive index of 2.61. Typically, it is encountered in the crystalline polymorphs of anatase, rutile, and brookite forms [28]. TiO_2_NPs have widespread applications due to their various shapes and specifically size-related features. Various physical, chemical, and biological synthesis techniques are employed in the synthesis of TiO_2_NPs (Fig. 1).

### 2.1 Physical synthesis method

Several physical synthesis techniques are widely used for the synthesis of TiO_2_NPs synthesis. Top-down or bottom-up techniques can be used to synthesize TiO_2_NPs. When using a top-down strategy, big structures are broken down into smaller ones. Among the most popular top-down approaches are physical techniques including lithography [29], laser ablation [30], sputtering deposition, and electrochemical etching [31]. Further, scalable manufacturing can be also fabricated by innovative methods including high-energy ball-milling [32], ultrasonication [33]and ultrasonic spray pyrolysis [34]. On the other side, nano-material is created atom by atom, molecule by molecule, or cluster by cluster in bottom-up methods [35]. The top-down method employs physical principles to break bulk TiO_2_ into nanoparticles. High-energy ball milling grinds bulk material into fine powder using small balls in a rotating container [36]. Lithography, a versatile technique, miniaturizes substrate material in a nanoscale range [37]. Laser Interference Lithography, combined with electrochemical anodization, creates hexagonally ordered TiO_2_ nanotubes. Metal-assisted chemical etching and nanosphere lithography fabricate Si/TiO2 core-shell nanopillar photoanodes [38]. TiO_2_ nanoparticles are produced by laser irradiation of a Ti target in water, with size distribution dependent on fluency. Sputtering, a physical approach, synthesizes TiO_2_ nanoparticles [39,40]. While physical methods demand advanced equipment and high costs, ball milling stands out for large-scale, low-cost, and non-toxic production [41]. The key drawbacks in physically synthesizing titanium dioxide nanoparticles involve difficulties in achieving precise size control, the potential for introducing impurities, and the need for sophisticated equipment, contributing to higher costs and limited scalability.

### 2.2 Chemical synthesis method

Various chemical synthesis techniques are largely employed for the synthesis of TiO_2_NPs such as sol-gel method [42], solvo-thermal method [43], co-precipitation method [44], hydrothermal method [45].While the chemical method for synthesizing TiO_2_ nanoparticles is commonly employed for its ease of synthesis and the ability to control the size and shape of nanoparticles, it has limitations related to cost-effectiveness, high temperature and pressure requirements, increased energy consumption, as well as concerns about eco-toxicity and environmental sustainability. Sol-gel process is widely used in TiO_2_ nanoparticles preparation, because it is simple straightforward and low-cost method. Sol-gel process involve in three steps, namely hydrolysis, condensation, drying and heat treatment steps. First, certain precursor of Ti such as TiCl_3_, Ti[OCH(CH_3_)_2_]_4_ (TTIP), TiCl_4_ and Ti(OBu)_4_ is dissolved in suitable solvent [46]. The TiO_2_ sol is formed through hydrolysis step which involve reaction of Ti precursor with water. The condensation is occurred by expelling the water and formation of TiO_2_ gel. The TiO_2_NPs is obtained by suitable dying and heat treatment of the TiO_2_ gel [47]. The dissociation of hydrolysis and condensation steps is important for controlling the crystallinity of TiO_2_ nanoparticles and can be enabled by adding acid, HCl, HNO_3_ or acetic acid [48]. In the precipitation method, Ti hydroxides react with basic agents like NaOH, NH_4_OH, and urea aqueous solutions, forming TiO_2_NPs of varied sizes upon calcination [49]. Hydrothermal synthesis in a closed system yields TiO_2_NPs, allowing control over particle size, shape, and distribution. Despite its effectiveness in tailoring nanostructures, this method is costly due to autoclave maintenance, time-consuming, and involves surfactants, potentially introducing impurities. Conversely, the solvo-thermal process, utilizing an autoclave at high temperature and pressure, is environmentally safe but expensive and complex [49]. The microemulsion method involves water, cyclohexane, and anionic surfactants, producing TiONPs with a minimum diameter of 20 nm through a hydrolysis reaction with titanium tetraisopropoxide (TTIP).

### 2.3 Biological sources

#### 2.3.1 Synthesis from green plants extract

Plant extracts are widely employed in TiO_2_NPs synthesis for their safety and efficacy. Components such as proteins, carbohydrates, enzymes, phenolic acids, and alkaloids within plants collaborate in synthesis-related reduction and stabilization processes. Leaves, with their rich metabolite content, are often preferred for green synthesis, leveraging their extracts for TiO_2_NPs production. According to Nabi et al. [50], cinnamon powder extract was used to generate TiO_2_NPs utilizing a green synthesis approach. It was determined that the synthesis procedure was simple, practicable, and inexpensive. It was found in another study that *Syzygium cumini* leaf extract may be used to synthesize both round and irregular TiO_2_ aggregated particles [51]. The technique was proved to be non-toxic, easy, economical, and environmentally favorable. Nevertheless, there are challenges and drawbacks associated with controlling size and morphology, potential variations in synthesis outcomes, and the requirement for optimized conditions to ensure consistent and reproducible results. Tetragonal TiO_2_NPs were successfully synthesized using extracts from *Moringa oleifera*, providing a simple and environmentally friendly method of synthesis [52]. Similarly, the utilization of *Trigonella foenum* leaves extract in the synthesis of TiO_2_NPs was found to yield a more environmentally friendly and time-efficient process [53]. In another study, pomegranate peel was utilized for the synthesis of TiO_2_NPs, which were used effectively for water disinfection without causing any harm to the environment [54].

#### 2.3.2 Synthesis from bacterial and fungal extract

The biosynthesis of TiO_2_NPs has also been carried out using both bacterial and fungal extract [55]. Bacterial biomass contains compounds that could aid in bio-reducing and stabilizing TiO_2_NPs. The microorganism *Streptomyces* sp. was utilized for the synthesis of TiO_2_NPs, resulting in a cost-effective, environmentally friendly, and pure product. The method demonstrated fast antimicrobial and antibiofilm activity [56]. Similarly, various bacteria such as *Aeromonas hydrophila* and *Lactobacillus* sp. were investigated for their potential in the simple, reproducible, and cost-effective synthesis of TiO_2_NPs [57,58]. Furthermore, compared to bacterial extract, fungal extract has received a lot of attention due to its benefits of easy extraction, mass manufacturing, viability from an economic standpoint, and enhanced surface of synthetic TiO_2_.Rajakumar et al. (2012) [59] employed the fungal species *Aspergillus flavus* as an innovative, beneficial, and environmentally friendly approach for the production of TiO_2_NPs through green synthesis. As fungi contain enzymes and metabolites that enable them to carry out the separation of bulk salts into elements of ions, various sizes and forms of TiO_2_NPs have been found [60].

### 2.4 Synthesis from other biological sources

Various biological sources beyond plants and microbes are employed in TiO_2_NPs production, offering economical and eco-friendly alternatives [60,61]. Starch is highlighted for its claimed ability to easily yield TiO_2_ with robust photocatalytic properties. Cellulose fibers efficiently produce homogeneous TiO_2_ nanowires, retaining fiber integrity [61]. Eggshells provide a biomimetic route for TiO_2_NPs synthesis, boasting high porosity and surface area. Egg albumen emerges as an eco-friendly, cost-effective, and reproducible medium for TiO_2_NPs production [62]. Another study utilizes egg albumen as a gelling agent in TiO_2_ manufacture. The low-cost, easy synthesis of TiO_2_NPs from starch further underscores its practicality in green synthesis [63].

## 3 TiO_2_NPs improve temperature stress tolerance in plants

**Fig. 2:**
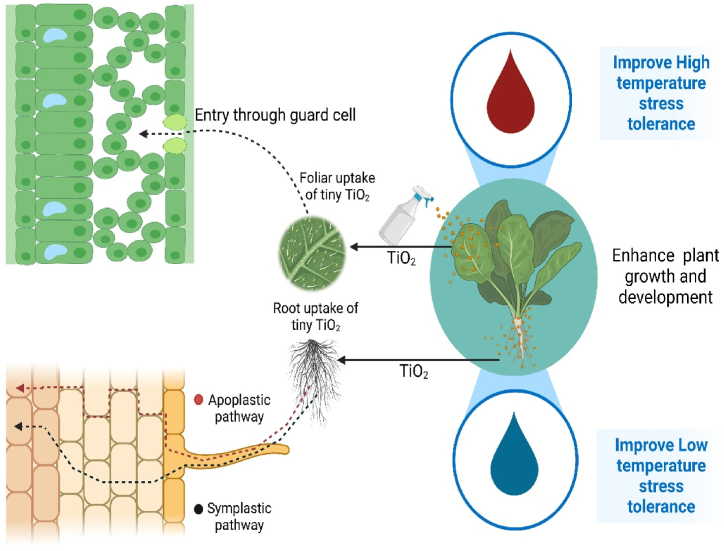
A schematic shows the TiO_2_NPs impact on the low and high temperature stress in plants. Created in Biorender.

With an increasing global population, the greatest challenge is to boost agricultural production [64]. In 2020, a staggering 811 million people experienced hunger, accounting for approximately one-tenth of the world's population [65]. In this situation, nanotechnology can be an extremely helpful tool for plant production. It has the potential to be employed in sustainable agriculture. When plants are treated with NPs (nanoparticles), the NPs are taken up by the plant through its roots or leaves [66]. The interaction between NPs and plants is influenced by factors like the chemical composition of the NPs, the structure of the plant's roots, and the properties of the soil [67]. The uptake, movement, and accumulation of TiO_2_NPs also depend on the size of the TiONPs. In general, NPs that are smaller than the cellular pores can easily enter the plant [68]. For example, NPs ranging from 4 to 100 nm can accumulate in the region below the stomata by disrupting the wax layer and cuticle [67]. Hence, it is evident that similar to other nanoparticles, tiny TiONPs can readily enter the plant through its leaves and roots (Fig. 2).

After being delivered into the plant, the TiO_2_NPs could move through two pathways: the apoplastic pathway and the symplastic pathway [68]. The apoplastic pathway allows the radial movement of nanoparticles, while the symplastic pathway enables movement within cells [65]. Through these pathways, TiONPs also can move to different parts of the plant and accumulate. The movement through the apoplastic pathway is governed by capillary forces and osmotic pressure, which may allow TiO_2_NPs to reach the endodermis beyond the cortex and epidermis. To enter the stele and associated vascular tissues, NPs need to use the symplastic route through aquaporins, endocytosis, ion channels, or carrier proteins. Once the NPs are internalized by cells, they can move to neighboring cells through plasmodesmata [65].

Furthermore, they have been applied to the roots or as a foliar spray to boost plant growth enzyme activity, chlorophyll content, photosynthesis, nutrient absorption, stress tolerance, yield, and crop quality. In addition, they provide favorable alternatives for farmers, compared with conventional chemicals and techniques, because of various properties such as their small size, simplicity of transport, easy handling, long-term storage, high efficacy, and nontoxicity. As a result, nano-based commercialization is gaining popularity worldwide. Because the environment is constantly changing, stress on plants comes from both biotic and abiotic factors. Plant growth and physiology could be affected by the stresses of both low and high temperatures.

### 3.1 TiO_2_NPs improve the low temperature stress tolerance in plants

Low temperatures (4 °C) have a substantial effect on plants, modifying their photosynthetic activity, biomass, and reactive oxygen species (ROS) generation at the cellular, physiological, and molecular levels [[69], [70], [71]]. MDA content serves as an indicator of the initial phases of membrane damage and can vary significantly based on the severity and duration of the treatment [12]. Exposure to low-temperature stress resulted in higher MDA content compared to control plants, indicating increased lipid peroxidation and membrane destruction, which induces oxidative stress in plants facing various challenges, including low-temperature stress [72]. Previous findings demonstrated that TiONPs reduced hydrogen peroxide and MDA levels as well as the electrolyte leakage index under low-temperature stress [12]. Excessive ROS production negatively affects DNA, proteins, and the membranes of cells [[73], [74], [75], [76]]. It is very important to control ROS generation and keep ROS levels stable [77,78]. The application of TiO_2_NPs results in an increase in the level of expression of genes that code for chlorophyll-binding and Rubisco proteins [13]. Amini et al. [79] used the cDNA-AFLP technique to demonstrate the impact of TiO_2_NPs on the development of low temperature stress tolerance in two chickpea genotypes (Table 1).

**Table 1:** Impact of TiO_2_ nanoparticles (TiO_2_NPs) on plant growth and development under low and high temperature stress.

Type of stress	Plant Species	TiO_2_ nanoparticles (TiO_2_NPs)	Temperature Stress level	Impact	References
Low temperature stress	*Cicer arietinum* L.*) genotypes,* cold sensi tive (ILC533) and cold_tolerant (Sel11439)	5 mg/L	4 °C	Decreased hydrogen peroxide and MDA contents and electrolyte leakage index (ELI)	[80]
	*Cicer arietinum* L.*) genotypes,* cold sensi tive (ILC533) and cold_tolerant (Sel11439)	0, 2, 5, and 10 ppm	4 °C	A significant decrease was observed in MDA content, electrolyte leakage index (ELI) at 5 ppm	[12]
	*Glycyrrhiza glabra* L.	2 and 5 ppm	4 °C		[81]
	Two *Cicer arietinum* L. genotypes (Sel96Th11439, cold tolerant, and ILC533, cold susceptible)	5 mgL-1	4 °C	Increased transcription levels of transcript-derived fragments (TDFs)	[79]
	*Cicer arietinum* L.) genotypes (Sel96Th11439, cold tolerant genotype, and ILC533, cold susceptible one)		4 °C	Increased the expression of genes encoding Rubisco- and chlorophyll-binding proteins, decreased H_2_O_2_ levels, and increased the activity of phosphoenolpyruvate carboxylase	[13]
High temperature Stress	*Lycopersicon esculentum* Mill.	T1 (0.05 g L^−1^), T2 (0.1 g L^−1^), and T3 (0.2 g L^−1^)	35 °C	Increased net photosynthetic rate, conductance to H_2_O, and transpiration rate, and photosystem II (PS II) energy dissipation	[14]
	*Triticum aestivum cv.* Unnat PBW 343 and *Triticum aestivum cv.* HD 2967	1.5 ppm and 10 ppm	32 °C	Increased superoxide dismutase (SOD). Catalase (CAT), and guaiacol peroxidase (GPX) activities. Decreased malondialdehyde (MDA) and hydrogen peroxide H_2_O_2_) contents	[25]

Cloning sixty (60) differentially expressed transcript-derived fragments (TDFs) from plants treated with TiO_2_NPs were cloned and ten (10) of them generated effectively readable sequences under low temperature stress [79]. Antioxidant enzymes, such as superoxide dismutase (SOD), catalase (CAT), ascorbate peroxidase (APX), guaiacol peroxidase (GPX), and polyphenol oxidase (PPO), have evolved in plants as a response to low temperature-induced oxidative stress. These enzymes are able to scavenge reactive oxygen species (ROS) [82]. Mohammadi et al. [80] found enhanced activity of antioxidant enzymes, which kept chlorophyll and carotenoids stable and improved resistance to cold stress. In addition, TiO_2_ NPs have a favorable effect on both susceptible (ILC 533) and resistant (Sel 11439) genotypes of chickpea when exposed to cold stress. It has been suggested that TiO_2_NPs promote cold tolerance in chickpea plants by boosting their protective mechanisms and lowering their injury levels. Future research could confirm the usefulness and processes of TiO_2_NPs in enhancing the cold tolerance of crops.

### 3.2 TiO_2_NPs improve the high temperature stress tolerance in plants

Crop performance has been significantly impacted by high temperatures, particularly in dry and semiarid areas [3]. Crops are particularly susceptible to the deleterious effects of high temperature stress on their essential morphological, physiological, and biochemical properties. Ultimately, it is abundantly clear that high temperature stress conditions cause plant entire systems to suffer. However, TiO_2_NPs have become increasingly used in recent years in agricultural fields that are exposed to high temperatures. The exogenous application of TiO_2_NPs noticeably alleviated the adverse effects of high-temperature stress and enhanced seedling growth. TiO_2_NPs treatment significantly increased root length compared to shoot length, indicating increased water uptake with supplementary effects in the immediate area rather than distant translocation [25]. Interestingly, it was observed that TiO_2_NPs improved seedling performance slightly better than other nanoparticles, such as zinc oxide (ZnO) nanoparticles [25]. Additionally, literature reports suggest that a concentration of 1.5 ppm of ZnO/TiO2 NPs is considered safe for crops [83]. Moreover, it was found that TiO2 NPs contribute to accelerating the seed germination rate [25]. Previous literature reports indicate that TiO_2_NPs generate reactive anions, such as superoxides and hydroxyl radicals [84], which directly or indirectly aid in water accumulation and increase the rate of oxygen uptake, essential for rapid seed germination. According to Mahmoud and Abdelhameed (2023) [85], plants treated with 15% TiO_2_NPs and carbon nanoparticles exhibited significant increases in seed yield and weight per 1000 seeds, with enhancements of 4.42 and 1.67 times, respectively. It was observed that spraying nanoparticles on sesame leaves led to improvements in the amino acid composition of sesame seeds [85]. This improvement was attributed to the physicochemical properties of nanomaterials (TiO_2_NPs), allowing them to enter cells through openings in the plasma membrane and induce changes in gene expression [86]. Additionally, the oil content in plants treated with 15% TiO_2_NPs and carbon nanoparticles increased by 48.99% compared to stressed plants. Moreover, under high-temperature stress, the proportion of unsaturated fatty acids in plants treated with TiO_2_NPs increased by 2.09 times compared to the control.

Similar to temperature stress, salt stress significantly decreased chlorophyll fluorescence parameters including Fv/Fm (a ratio that indicates the quantum efficiency of photosystem II: maximal quantum yield of PSII), Fv/Fo (a parameter that accounts for the simultaneous variations in Fm and Fo in determinations of the maximum quantum yield of PS II: Efficiency of the water-splitting complex on the donor side of PSII) and Y(II) (the complementary quantum yields of PS II). It was found that TiO_2_NPs application improved chlorophyll fluorescence parameters under stress conditions [87,88]. Under both control and stress conditions, all TiO_2_ NP applications increased Fv/Fm values. The current findings suggest that TiO_2_ NPs could ameliorate temperature stress by improving chlorophyll fluorescence parameters and maximizing PSII efficiency.

Plant responses to high temperatures have a reduction in photosynthesis capacity and the quantity of photosynthetic pigments in leaves [2,3]. Application of TiO_2_NPs had significant effects on photosynthesis pigments [88].Extreme temperatures quickly cause leaves to decrease their water content and weaken the rigidity of their membranes [89]. They also influence the mechanisms of photosynthetic rate [90]. Rubisco is one of the photosynthetic enzymes that can be damaged by high temperatures. These enzymes actively contribute to the process of CO_2_ fixation [91]. Temperature variations have an effect on both the rates of ribulose-1,5-bisphosphate (RuBP) production and the carboxylation sites of Rubisco [92]. Qi et al. [14] demonstrated that the use of TiO_2_NPs boosted the net photosynthetic rate, stomatal conductance, and transpiration rate of tomato leaves. These findings suggest that TiO_2_NPs have a beneficial effect on photosynthetic improvement in plants under high temperature stress.

Malondialdehyde (MDA) functions as a biomarker for assessing membrane stability by revealing oxidative damage incurred by the membrane due to high-temperature stress [93]. Conversely, hydrogen peroxide serves as a crucial, relatively stable non-radical reactive oxygen species (ROS) produced during normal plant metabolism. The excessive accumulation of H_2_O_2_ can induce oxidative stress and eventual cell death in plants. The application of nanoparticles resulted in a reduction in both MDA and H_2_O_2_ levels. This implies that TiO_2_NPs potentially mitigate high-temperature stress through a mechanism involving membrane repair. It is plausible that TiO_2_NPs pretreatment leads to a decrease in ion leakage in grains, thereby enhancing cell membrane integrity [25]. Plants have developed a complex enzymatic system and non-enzymatic antioxidants that assist in scavenging these naturally produced reactive oxygen species (ROS) induced by high temperature stress [94,95]. The application of nanoparticles at the proper dosage modifies the activity of antioxidant enzymes, hence enhancing their resistance to high-temperature stress. Increased ROS generation impairs cell viability through lipid peroxidation, membrane degradation, and enzyme inactivation. Pretreatment of TiO_2_NPs considerably increased tolerance to high temperature stress by increasing SOD and GPX activity, resulting in an additional decrease in H_2_O_2_ concentrations [25].

## 4 Research gaps

In order to understand the protective effects of beneficial TiO_2_NPs in mitigating the adverse effects of temperature stress on plants, there are still numerous research gaps that need to be addressed. While it has been reported that TiO_2_NPs can alleviate temperature stress in plants, there is a need for comprehensive investigations to identify and study the involvement of temperature stress-related genes (regulated by TiO_2_NPs) at the molecular level. Limited studies have been conducted on the molecular basis of TiO_2_NPs in enhancing temperature stress tolerance and promoting plant growth characteristics. Exploring these aspects could enable plant scientists and farmers to develop effective TiO_2_NPs that can alleviate other abiotic stresses and contribute to sustainable agriculture. The mechanisms by which TiO_2_NPs enter and move within aerial plant parts, particularly grains, are still unclear. It is necessary to confirm the effectiveness of various application methods for TiO_2_NPs, such as seed priming, root exposure in nutrient solutions, and foliar spray, in managing the toxicity induced by low and high temperatures. Furthermore, it is crucial to investigate the impacts, both positive and negative, of TiO_2_NPs on the different stages of the plant's life cycle under temperature stress and during long-term exposure in both greenhouse and field conditions.

## 5 Future prospect

The potential of nanotechnology to enhance plant performance by improving tolerance to biotic and abiotic stresses has garnered significant attention in recent years within the agro-food system. It has been proven that exogenous application of TiO_2_NPs to a variety of species increases plant tolerance to temperature stress. Moreover, the above studies provide clear evidence that TiO_2_NPs positively impact the photosynthetic apparatus and enhance photosynthesis in plants exposed to stressful conditions. Consequently, the incorporation of TiO_2_NPs in future practices becomes imperative in order to enhance the health and sustainability of plant species. These advantages justify using the appropriate amount of TiO_2_NPs to improve agricultural yields. Plant biotechnologists are interested in TiO_2_NPs because to their potential environmental benefits, and it is believed that they could be a helpful method for addressing issues with plant development and yield that are now limiting agricultural advancement. The crop yield might increase if these issues are fixed. The positive and negative impacts of TiO_2_NPs on plants remain unclear due to a lack of understanding of the molecular mechanism behind these effects. Because of this, the connection between the characteristics of TiO_2_NPs and the expression of the genes and proteins necessary for the plant's development remains unknown. It is recommended that future research look into how the physicochemical properties of TiO_2_NPs play a role in their translocation into the plant organs via the root system.

## 6 Conclusion

On a global scale, crop production has encountered numerous challenges stemming from climatic conditions and various stresses. Nanotechnology has emerged as a pivotal facet in promoting environmental sustainability, aiming to effectively address and overcome these hurdles. In this study, we focused on the newest findings on the use of TiO_2_NPs in various species that were exposed to some of the most frequent climatic factors, like temperature. Numerous studies conducted under controlled and field situations have clearly shown the positive benefits of TiO_2_NPs on plants exposed to low and high temperature stresses at the metabolic and physiological levels. These effects could be influenced by a number of factors, including the form of NP applied and how it was applied, the dosage, and the kind and degree of stress exposure. In general, the utilization of these TiO_2_NPs possesses the potential to increase plant productivity and may serve as a sustainable, enduring remedy for the issues arising from abiotic stressors in agricultural species. Despite their limitations, TiO_2_NPs exhibit significant promise as a cost-effective strategy to bolster crop productivity and enhance resistance to abiotic stress in the forthcoming decades.

## Additional information

No additional information is available for this paper.

## CRediT authorship contribution statement

**Nadiyah M. Alabdallah:** Writing – original draft, Validation, Supervision, Project administration, Funding acquisition, Formal analysis, Conceptualization. **Saleh M. Alluqmani:** Writing – review & editing, Supervision, Software, Resources, Project administration, Methodology, Formal analysis, Data curation. **Hana Mohammed Almarri:** Writing – review & editing, Writing – original draft, Visualization, Methodology, Investigation, Conceptualization. **Asla A. AL-Zahrani:** Validation, Methodology, Formal analysis, Data curation.

## Declaration of competing interest

The authors declare that they have no known competing financial interests or personal relationships that could have appeared to influence the work reported in this paper.
